# Effect of π–π Stacking Interfacial Interaction on the Properties of Graphene/Poly(styrene-*b*-isoprene-*b*-styrene) Composites

**DOI:** 10.3390/nano11092158

**Published:** 2021-08-24

**Authors:** Xiaobing Han, Hao Kong, Tao Chen, Jie Gao, Yuan Zhao, Yanan Sang, Guowen Hu

**Affiliations:** Hubei Key Laboratory of Radiation Chemistry and Functional Materials, School of Nuclear Technology and Chemistry & Biology, Hubei University of Science and Technology, Xianning 437100, China; hanxiaobing@hbust.edu.cn (X.H.); hbustchemistry@163.com (H.K.); taochen518@163.com (T.C.); zhyf308@hbust.edu.cn (Y.Z.); sangyanan2021@163.com (Y.S.)

**Keywords:** interfacial interaction, graphene, polymer composite, properties

## Abstract

Interfacial interaction is one of the most important factors in the construction of high-performance graphene-based elastomer composites. In this paper, graphene/poly (styrene-*b*-isoprene-*b*-styrene) (SIS) composites were prepared with solution mixing followed by an evaporation-induced self-assembly process. Various techniques such as scanning electron microscopy, UV-vis absorption spectra, tensile testing, Shore A hardness, surface resistance, thermal conductivity, and thermogravimetric analysis were conducted to characterize the microstructure and properties of the obtained composites. The results showed that the π–π stacking interfacial interaction between phenyl groups of SIS and graphene play an important role in the properties’ improvement, and the effect of interfacial interaction on the properties was revealed.

## 1. Introduction

Polymer composites based on nanofillers such as fullerene, carbon nanotubes, nano-diamond, and layered silicates have attracted much attention in recent years, because of their prominent properties and numerous applications [1,2,3]. Nowadays, graphene, a multifunctional nanofiller, has been revealed to be a promising reinforcing component for the construction of polymer composites [4,5,6]. To maximize the reinforcing efficiency of graphene in polymer composites, the issues of dispersion state and interfacial interaction between graphene and polymer matrix should be addressed [7,8,9]. Many methods were developed for the fabrication of graphene-based polymer composites, such as solution mixing, melt blending, and in situ polymerization. Among these methods, solution mixing has been demonstrated to be an effective way to obtain satisfactory dispersion [10,11,12].

The other important factor that influence the properties of graphene-based polymer composites is the interfacial interaction, which determines the stress dispersion and transport performance of the obtained nanocomposites [10,11,12,13,14]. As reported by the group of Wang [15], the interfacial orientation of the polystyrene phenyl groups in contact with graphene was revealed by sum frequency generation vibration spectroscopy. The phenyl groups prefer to recline to more favorably interact with graphene via a face-to-face configuration (π–π stacking) at a low concentration; this provides important knowledge for the design and optimization of graphene-based aromatic polymer nanocomposites. Guo et al. reported the rational design of covalent interfaces for graphene/styrene butadiene rubber nanocomposites [16]; the strong interfacial interaction (π–π stacking, hydrogen bond, covalent crosslinking) provides synergetic improvement in the mechanical properties (drastically decreased energy loss). Zhong et al. reported the influences of reduced graphene oxide (RGO) on the interfacial interaction and electrical conductivity of polycarbonate (PC) [17]; the nanosized effect, good conductivity of RGO, and strong interfacial interaction (π–π stacking) between RGO and PC resulted in a low conductive threshold of 0.36 wt%. Wang et al. reported the effect of RGO on the thermal conductivity of poly (vinyl alcohol) (PVA) composites [18]; the thermal conductivity of the composites was closely associated with interfacial interaction: the stronger the interfacial interaction, the larger the thermal resistance and the lower of thermal conductivity. Hofmann et al. reported that for thermoplasctic SEBS nanocomposites reinforced with functionalized graphene [19], enhanced gas barrier resistance was attributed to the labyrinth effect (drastically increasing the diffusion pathway) and strong interfacial interaction (π–π stacking) between graphene and SEBS. Though the interfacial interaction plays an important role in the construction of graphene-based polymer composites, there are few systematic reports about the effect of the interfacial interaction on different properties for specific polymer composites [20].

Styrene-based block copolymers are basically thermoplastic elastomers, with good performance for a wide range of applications, such as toys, packaging, adhesive, and medicinal materials [21]. Poly (styrene-*b*-isoprene-*b*-styrene) (SIS) is a triblock copolymer belonging to the family of thermoplastic elastomers. The SIS composites with carbon nanotube or graphene have applications in photomechanical actuation [22,23]; however, there are few reports about the effect of interfacial interaction on different properties of SIS composites. In the present work, the SIS doped with different loadings of graphene were prepared with solution mixing followed by an evaporation-induced self-assembly process, enhanced properties were obtained through a π–π stacking interfacial interaction, and the effect of interfacial interaction on the properties was revealed.

## 2. Materials and Methods

### 2.1. Materials

Graphene was purchased from Suzhou TanFeng Graphene Tech Co., Ltd. (Suzhou, China). Poly (styrene-*b*-isoprene-*b*-styrene) (Melt flow rate = 23 g/10 min) was purchased from Xingzhi New Materials Co. Ltd. (Guangzhou, China). Chloroform was obtained from Sinopharm Chemical Reagent Co., Ltd. (Shanghai, China) without purification.

### 2.2. Preparation of Graphene/SIS Composites

Graphene/SIS nanocomposites with different graphene concentrations (0~2.0 wt%) were prepared by a solution mixing method. In a typical procedure, the graphene was dispersed into 10 mL chloroform using a bath sonicator for 1 h. Then, 4 g of SIS was added into the dispersion with vigorous stirring for 2 h, and the mixture was sonicated for 1 h. Finally, the obtained mixture was poured into a polytetrafluoroethylene mold, the graphene/SIS films were obtained with an evaporation-induced self-assembly process, and the obtained films were dried at 60 °C to a constant weight.

### 2.3. Characterization

Scanning electron microscopy (SEM, VEGA 3SBH, Tescan Orsay Holding, Brno, Czech Republic) was used to examine the morphology of the graphene sheets. UV-Vis absorption spectra were conducted with a S 3100 spectrophotometer (Mapada Instruments Co. Ltd., Shanghai, China). The tensile properties of graphene/SIS composites were measured by a universal testing machine (Shimadzu AG-IC, Zhujin Analytic Instruments Co. Ltd., Shanghai, China); at least five samples were tested to obtain average values. Measurements of Shore A hardness consisted of vertical immersion of the indenter into the composite surface with a Shore hardness tester (LX-A, Shenzhen Haoxinda Instrument Co., Ltd., Shenzhen, China). The surface resistance of the obtained composites was measured with an ultra-high resistance micro-current tester (ST2643, Suzhou Jingge Electronic Co., Ltd., Suzhou, China). The thermal conductivity of the composites was measured using thermal conductivity test equipment (DRE-2C, Xiangtan Instrument Co., Ltd., Xiangtan, China). Thermogravimeter (TG) analysis was performed with a TG-209-F3 (PerkinElmer, Waltham, MA, USA) under the nitrogen atmosphere at a heating rate of 10 °C/min from 30 to 650 °C.

## 3. Results

### 3.1. Morphology of Graphene

**Figure 1:**
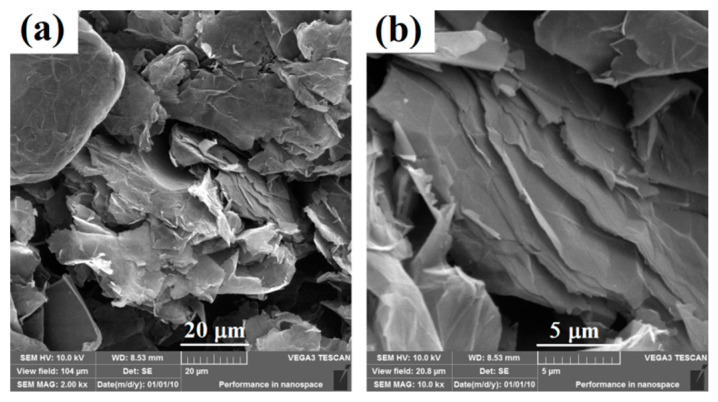
SEM images of graphene with (**a**) low resolution and (**b**) high resolution.

Scanning electron microscopy (SEM) of the graphene powder is given in Figure 1, which appears to be a rigid layered structure and very large in size. The edges of the graphene nanosheets are well defined with sharp corners, which is consistent with the results in the literature [11,23]. The large size of graphene sheets can provide sufficient surface area for the adhesion of polymer. Furthermore, strong interfacial interaction (π–π stacking) can be formed between graphene and aromatic polymer nanocomposites, resulting in improved properties. 

### 3.2. π–π Stacking Interaction between Graphene and SIS

**Figure 2:**
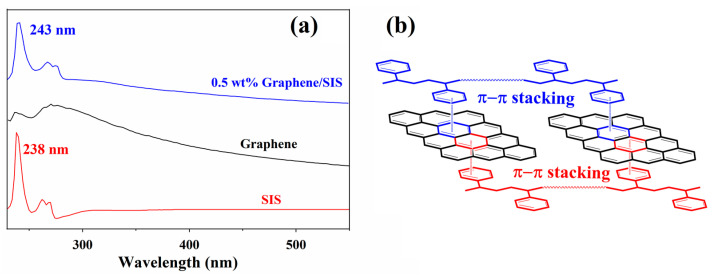
(**a**) UV absorption spectra and (**b**) π–π stacking interactions between graphene and SIS.

Solution mixing has been found to be an effective method for the preparation of graphene-based polymer composites, as the polymer can be absorbed on the surface of graphene through interfacial interaction, resulting in a good dispersion state [11,12]. The dried graphene/SIS composite was redissolved into chloroform, and UV-vis absorption spectroscopy was carried out to confirm the interfacial interaction between graphene and SIS in the composites. As shown in Figure 2a, the spectrum of pristine graphene exhibits a broad absorption peak at 270 nm and a shoulder peak at 236 nm. The spectrum of pure SIS exhibits a sharp absorption peak at 238 nm, originating from the polystyrene block in this block copolymer, whereas that of 0.5 wt% graphene/SIS composite exhibits a sharp peak at 243 nm. The peak of composite shifts to a higher field by 5 nm compared to that of pure SIS, originating from the effect of ring currents in graphene and polystyrene block π-systems; this can be ascribed to the formation of nocovalent π–π stacking between phenyl groups of SIS chains and basal planes of graphene (Figure 2b) [24,25]. These results demonstrate that a π–π stacking interaction can be formed in the slow self-assembly process; thus, the solution mixing followed by evaporation-induced self-assembly process is an effective method to obtain strong interfacial interaction [24,25]. These strong interfaces can facilitate stress dispersion and transport performance [10,11,12,13,14], which has an important effect on the properties of the graphene/SIS composites.

### 3.3. Mechanical Properties of Graphene/SIS Composites

**Figure 3:**
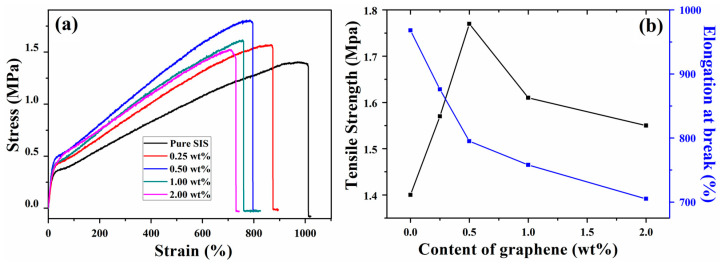
Stress–strain curves (**a**) and mechanical properties (**b**) of graphene/SIS composites.

Tensile testing was used to evaluate the mechanical properties of pure SIS and the graphene/SIS composites. The stress–strain behavior of all samples is presented in Figure 3a. It can be seen that with the incorporation of graphene, the tensile strength of the obtained composites improved significantly, compared to the pure SIS. The tensile strength and elongation at break are also plotted against graphene content in Figure 3b. Compared with pure SIS, the tensile strength of the composites increased at a low content of graphene: the tensile strength of the 0.5 wt% graphene/SIS composites increases by 26.4% from 1.4 to 1.77 MPa. Then, the tensile strength consistently decreases when the graphene content exceeds 0.5 wt%, but it remains higher than that of pure SIS [26]. This may be ascribed to the different dispersed state and interfacial interaction of graphene in SIS matrix. Graphene was fully exfoliated at low content and exhibited strong a π–π stacking interaction with the SIS chain, and the π–π stacking interaction can serve as a sacrificial bond to dissipate energy, leading to the effective dissipation of stress. On the contrary, the aggregation of graphene at relatively high contents exhibits weak interfacial interaction, leading to a poor dissipation of stress [27,28]. The elongation at break declines significantly with the increasing of graphene content, and a similar phenomenon was also observed not only for thermoplastic elastomer composites, but also for most polymer composites where agglomerated fillers act as failure points during elongation [19,29,30].

### 3.4. Shore A Hardness of Graphene/SIS Composites

**Figure 4:**
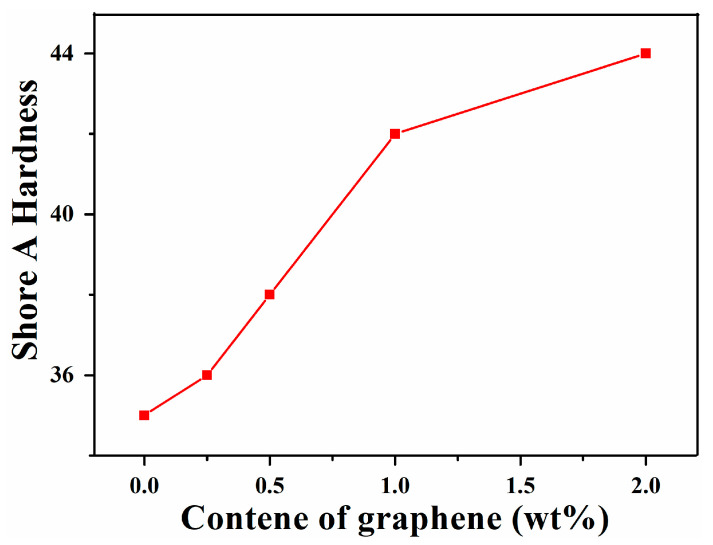
Shore A hardness of SIS and graphene/SIS composites.

The investigation of Shore A hardness of SIS and corresponding graphene nanocomposites as a function of filler content are shown in Figure 4. It is clear that the hardness value of the graphene/SIS nanocomposites shows an increasing trend with increase in graphene content in the polymer matrix. The maximum hardness value recorded is about 44 for the composite with 2 wt% of graphene, which corresponds to an improvement of 25.7% compared to that of unreinforced SIS. This phenomenon arises from the addition of rigid graphene nanosheet, which is denser and harder than SIS matrix. It is also observed that the increase in hardness value is smaller when the content of graphene exceeds 1 wt%, which indicates that the surface of the nanocomposites becomes more homogeneous and harder [19,30,31]. 

### 3.5. Surface Resistivity of Graphene/SIS Composites

**Figure 5:**
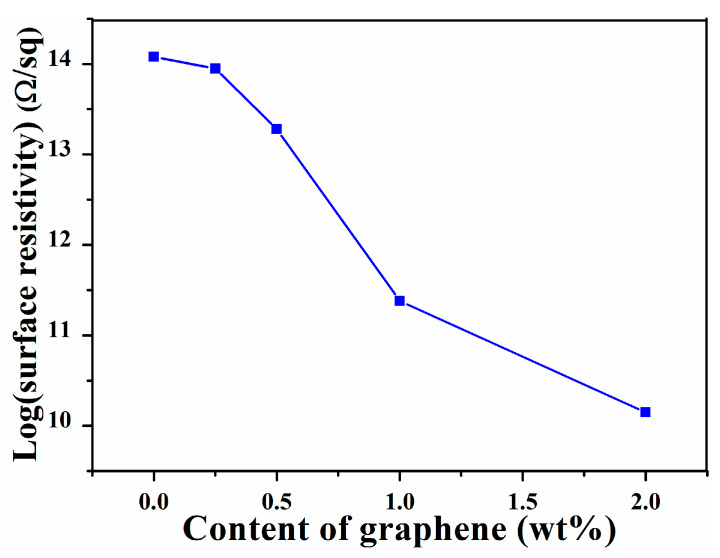
Surface resistivity of SIS and graphene/SIS composites.

According to the electrical percolation theory, graphene sheets provide percolated pathways for electron transfer, which imparts electric conductivity to the nanocomposites. However, the improvement efficiency of graphene sheets dramatically depends on the degree of sheets’ dispersion and interfacial interaction [32,33]. The electrical properties of graphene/SIS composites obtained with different contents of graphene were investigated in detail. The dependence of electrical property on graphene sheet loading is present in Figure 5. The surface resistivity decreased slowly when the content of graphene was below 0.5 wt%, which can be ascribed to the good dispersal of graphene sheets and the fact that the conductive network was not formed. The surface resistivity decreased sharply when the content of graphene exceeded 0.5 wt%, and the surface resistivity of 2.0 wt% graphene/SIS composite decreased by four orders of magnitude. This is due to the formation of a conductive network and the π–π stacking between graphene and SIS [33,34]. 

### 3.6. Thermal Conductivity of Graphene/SIS Composites

**Figure 6:**
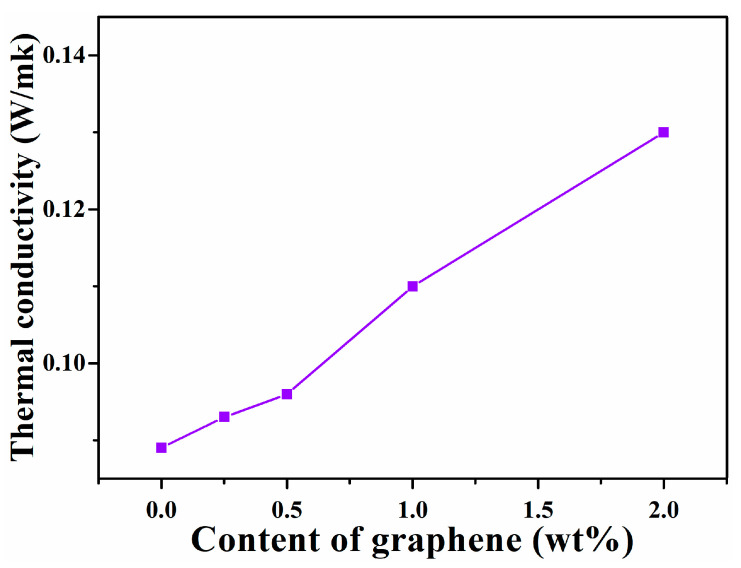
Thermal conductivity of SIS and graphene/SIS composites.

Thermally conductive polymer composites are attracting considerable attention, especially in recent years because increasingly more powerful electronics are being developed [35]. The thermal conductivity of SIS and SIS composites are summarized in Figure 6. The unfilled SIS has the lowest value of 0.089 W/mK. The thermal conductivity of the graphene/SIS composites increased with the increase in graphene content, the highest increase with respect to the unfilled SIS was for the 2.0 wt% graphene/SIS composite, and was 42%, which can be ascribed to the high thermal conductivity of graphene [35,36,37]. The interfacial interaction between nanofiller and polymer has an important effect on the thermal conductivity, which dominates the delivery of phonons between matrix and nanofillers [38,39]. When the content of graphene was below 0.5 wt%, the thermal conductivity of the graphene/SIS composites deviates from linear growth, which is due to the π–π stacking interaction between graphene and SIS. At low content, the graphene presents a well exfoliated state; the high specific surface area and interfacial interaction provide more sites, which can scatter phonons and damp the vibrational amplitude at the interface, inducing a higher thermal resistance. As the content of graphene sheet is increased, the graphene agglomerates and the interfacial interaction-induced thermal resistance is limited [18,39]. 

### 3.7. Thermal Stability of Graphene/SIS Composites

**Figure 7:**
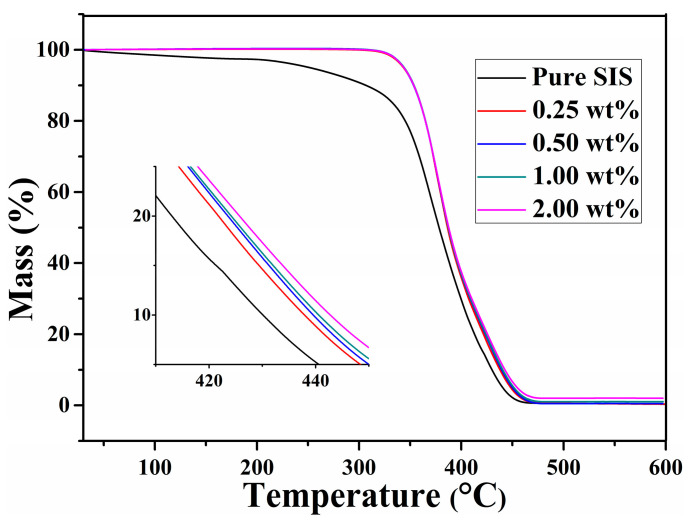
TG curves of SIS and graphene/SIS composites.

Increased thermal stability is typical of polymer-layered nanocomposites, usually attributed to heat and mass barrier effects of layered nanocompounds, which delay heat and pyrolysis products’ diffusion. The thermal stability of graphene/SIS composites is presented in Figure 7. The pure SIS shows a slow thermal degradation at a temperature range of 30–350 °C, which becomes more dramatic after 350 °C, before being almost completely decomposed at 450 °C [23]. It is found that all the composites present similar degradation behavior to pristine SIS, and the composites have an obvious improved thermal stability in comparison to pure SIS, especially in the range of 30–350 °C. This improvement in thermal stability could be attributed to the tortuous path effect, which formed between graphene and SIS through π–π stacking [10,40]. The decreased degradation rate with increase in graphene content can be ascribed to the effective obstruction of low molecules from degraded SIS, and the shield function of the heat [32]. 

## 4. Conclusions

In summary, the graphene/SIS composites were prepared through solution mixing followed by an evaporation-induced self-assembly process, enhanced mechanical, electrical, thermal properties were obtained through π–π stacking interfacial interaction, and the effect of interfacial interaction on the properties was revealed. The results of UV-vis absorption spectra revealed that π–π stacking interaction between phenyl groups of SIS and graphene can be formed in the process of evaporation-induced self-assembly. The π–π stacking interaction can serve as a sacrificial bond to dissipate energy, leading to the effective dissipation of stress, and the tensile strength of the 0.5 wt% graphene/SIS composites is increased by 26.4% from 1.4 to 1.77 MPa. The π–π stacking interaction benefits from the formation of a conductive network; the surface resistivity of 2.0 wt% graphene/SIS composite decreased by four orders of magnitude. The high specific surface area and π–π stacking interaction provide more sites that can scatter phonons and damp the vibrational amplitude at the interface, inducing a higher thermal resistance. The π–π stacking interaction is benefits for the effective obstruction of low molecules from the degraded SIS and the shield function of the heat; thus, the obtained composites exhibit improved thermal stability in comparison to pure SIS.

## Data Availability

Data is contained within the article.
